# Development and Clinical Validation of a Multiplex Real-Time Quantitative PCR Assay for Human Infection by *Anaplasma phagocytophilum* and *Ehrlichia chaffeensis*

**DOI:** 10.3390/tropicalmed3010014

**Published:** 2018-01-29

**Authors:** Megan E. Reller, J. Stephen Dumler

**Affiliations:** 1 Division of Infectious Diseases, Department of Medicine, Duke University School of Medicine, Durham, NC 27710, USA; 2 Duke Hubert-Yeargan Center for Global Health, Durham, NC 27708, USA; 3 Duke Global Health Institute, Durham, NC 27710, USA; 4 Joint Departments of Pathology, F. Edward Hebert School of Medicine, Uniformed Services University of the Health Sciences, Walter Reed National Military Medical Center, Joint Pathology Center, Bethesda, MD 20814, USA; john.dumler@usuhs.edu

**Keywords:** *Ehrlichia chaffeensis*, *Anaplasma phagocytophilum*, Rickettsiales, ticks, diagnostics, etiology of fever studies

## Abstract

Background: Human granulocytic anaplasmosis (HGA), caused by *Anaplasma phagocytophilum*, and human monocytic ehrlichiosis (HME), caused by *Ehrlichia chaffeensis*, often present as undifferentiated fever but are not treated by typical empiric regimens for acute febrile illness. Their role as agents of vector-borne febrile disease in tropical regions is more poorly studied than for other rickettsial infections. Limitations in diagnosis have impaired epidemiologic and clinical research and needless morbidity and mortality occur due to untreated illness. Methods: We designed and clinically validated a multiplex real-time quantitative PCR assay for *Anaplasma phagocytophilum* and *Ehrlichia chaffeensis* using samples confirmed by multiple gold-standard methods. Results: Clinical sensitivity and specificity for *A. phagocytophilum* were 100% (39/39) and 100% (143/143), respectively, and for *E. chaffeensis* 95% (20/21) and 99% (159/161), respectively. Conclusions: These assays could support early diagnosis and treatment as well as the high-throughput testing required for large epidemiologic studies.

## 1. Introduction

Human granulocytic anaplasmosis (HGA), caused by *Anaplasma phagocytophilum*, and human monocytic ehrlichiosis (HME), caused by *Ehrlichia chaffeensis,* often present as undifferentiated fever [1,2]. Not only are these infections difficult to recognize clinically, they are also difficult to diagnose. Further, they require specific treatment (doxycycline) that is not used for empiric treatment of acute febrile illness.As advanced diagnostic methods are employed to identify etiologic agents of undifferentiated fever, rickettsial infections are increasingly recognized causes of febrile illness in the tropics with substantive morbidity and mortality [3]. Despite the increasing recognition of infections by *Rickettsia* and *Orientia* species in the Rickettsiaceae family, only limited study of human infection by Anaplasmataceae has been conducted in tropical regions [4,5,6,7,8,9,10]. This sustained lack of progress occurs despite clear evidence of the presence of *Anaplasma*, *Ehrlichia*, and *Neorickettsia* species in vectors and as agents of disease in wild and domestic animals. Limitations in diagnosis impair clinical diagnosis of these infections in humans and clinical and epidemiologic studies of these illnesses. Treatment remains empiric. Therefore, we developed a multiplex real-time PCR assay to rapidly detect and distinguish these pathogens in acutely ill patients.

## 2. Results

Clinical validation was accomplished with 13 and 39 blood samples for the singleplex and multiplex qPCR assays, respectively, from 42 patients with confirmed HGA. For HME, we used six and 21 blood samples from 23 patients with HME; 13 samples were provided as extracted DNA by J. Olano and J. McBride (University of Texas, Galveston, TX, USA). Confirmation included demonstration of morulae on peripheral blood smear, culture, alternative-target PCR, and/or a 4-fold rise in IgG titer in paired sera, and by 2 or more gold-standard methods in all but three [11,12]. For the singleplex assays, specificity was assessed by testing blood from 20 patients with confirmed typhus group rickettsiosis, eight with spotted fever group rickettsiosis, seven with scrub typhus, one convalescent from HGA, three convalescent from HME, 22 with malaria, and one with confirmed non-*Ehrlichia/Anaplasma* bacteraemia. In addition, for HGA specificity, all six samples from patients with HME were tested, whereas for HME specificity, all 13 samples from patients with HGA samples were tested. *ACTB* amplification from all samples was detected.

For the multiplex qPCR assay, specificity was assessed using 78 blood samples from patients with malaria (45 *Plasmodium falciparum*, 22 *P. vivax*, 11 *Plasmodium* spp.), two with *Babesia* spp., eight with bacteraemia (four *S. aureus*, one *Pseudomonas fluorescens*/*putida*, one *Enterococcus* non-*faecalis*, one *E. coli*, and one *Enterococcus* spp.), one convalescent from HME, and 33 from hospitalized patients with no identified infection.For HGA, eight samples from *E. chaffeensis*-infected patients were also used to assess specificity. Thirty-nine samples from patients with HGA were used to assess *E. chaffeensis* multiplex qPCR specificity.

**Figure 1:**
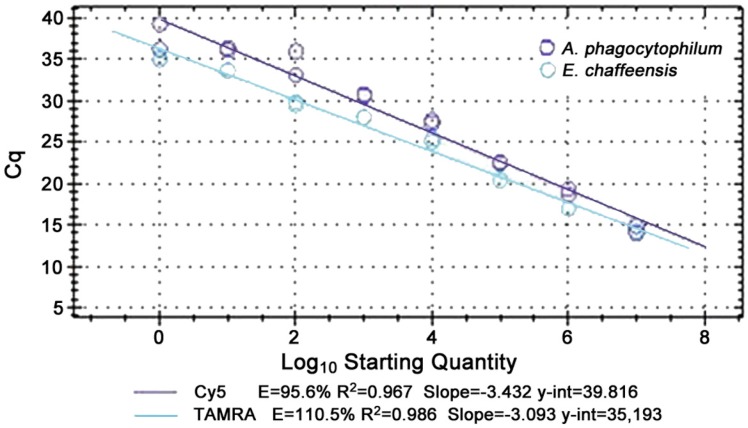
Standard curves for *A. phagocytophilum* (Cy5) and *E. chaffeensis* (TAMRA) in the multiplex qPCR. Note similar efficiencies and linearity from 10^7^ to 10^0^ copies.

Analytical sensitivity was as low as one copy (<1 organism/μL blood) for *A. phagocytophilum* and *E. chaffeensi*s, and analytical specificity was 100% for both singleplex and multiplex assays. The multiplex reaction was linear from 10^7^ to 10^0^ copies for all targets with replicate runs and efficiencies of 85 to 115% were achieved with R^2^ values usually above 0.95 (Figure 1). Analysis of receiver operator characteristic (ROC) curves for the multiplex qPCR assay identified mean relative fluorescence units (RFU) of negative controls plus 10% of high RFU-low RFU per plate as the optimal cut-off for *A. phagocytophilum* and 3% as best for *E. chaffeensis*.

The clinical sensitivity of the singleplex qPCR for *A. phagocytophilum* was 92% (12/13 *A. phagocytophilum*) and specificity 100%. For the *E. chaffeensis* singleplex qPCR, sensitivity was 83% (5/6 *E. chaffeensis*) and specificity was 100%. The multiplex clinical sensitivity and specificity for *A. phagocytophilum* were 100% (39/39) and 100% (130/130 with other infections or no identified infection), respectively. The multiplex’s clinical sensitivity and specificity for *E. chaffeensis* was 95% (20/21) and specificity 99% (160/161 with other infections or no identified infection). The assays could be performed in <3 h. The single false positive result at these cutoffs was found when testing for *E. chaffeensis* in a sample from a patient with HGA. Of the 39 samples from patients with *A. phagocytophilum* tested, the median bacterial load was 1723/μL (IQR 436 to 8885; min 0.04, max 290,857) and for the 21 from those with *E. chaffeensis*, the median bacterial load was 1929/μL (IQR 21 to 12,204; min 2, max 131,835).

## 3. Discussion

HGA and HME are globally-distributed, life-threatening tick-borne rickettsial diseases [13] that present as undifferentiated fever but are not treated by typical empiric regimens for acute febrile illness. Clinically suspected HGA and HME with supportive serologic and occasional molecular evidence have been reported in humans in Europe, North and South America, Africa, and the Far East, including China and Korea. We documented *Ehrlichia chaffeensis* as a cause of acute febrile illness in Nicaragua [6]. To define a confirmed case of HGA or HME, the European Society of Clinical Microbiology and Infectious Diseases and the Centers for Disease Control and Prevention require 1) a 4-fold increase in IgG titer by indirect immunofluorescence assay (IFA) between paired (acute and convalescent) sera, since testing acute phase sera alone is neither sensitive nor specific, or 2) direct detection of the pathogen by PCR or cell culture [14,15]. Diagnosis by IFA using paired sera is infrequently accomplished and is inherently retrospective, subjective, and time-consuming. Screening convalescent sera using a peptide-based ELISA and confirming positives by IFA [6] improves throughput and objectivity to better support large clinical studies but still cannot confirm diagnoses in real-time, as is required for patient care. Further, serologic cross-reactions commonly (3–30%) preclude species-level diagnosis [16]. Culture is slow and not widely available.Although other molecular assays for Anaplasmataceae have been reported [12,17,18,19], we uniquely describe extensive clinical validation with samples from patients in whom the targeted pathogens were confirmed by two or more gold-standard methods. Further, we used the same conditions we used for detection of other clinical mimics [20,21,22,23] in the tropics to allow adaptation of the assay to detect other geographically-relevant causes of undifferentiated fever, as shown here by high specificity when samples from patients with *E. chaffeensis* and *A. phagocytophilum* were tested for spotted fever or typhus rickettsioses, scrub typhus, malaria, and babesiosis, as well as other bacteraemias. Our real-time quantitative qPCR assay could support needed epidemiologic assessments of the global distribution of *A. phagocytophilum* and *E. chaffeensis* and earlier diagnosis and treatment, both required to reduce morbidity and mortality from these diseases worldwide.

## 4. Materials and Methods

We designed a singleplex 5′ nuclease quantitative PCR (qPCR) assay that targets *A. phagocytophilum* multicopy (10^2^ copies per genome) *msp2*. We adapted the assay to simultaneously detect and distinguish *E. chaffeensis* using the same conditions we have used for other targets, including *Rickettsia*, *Orientia*, *Borrelia,* and malaria [20,21]. AlleleID^®^ v 6.1 software (Premier Biosoft International, Palo Alto, CA, USA) was used to design primers and probes using standardized parameters (18–25 base-pair primer length; 59 ± 5 °C primer Tm; 100 mM monovalent ions; 5.0 mM free Mg^+2^; and cycling at 95 °C for 3 min, 40 cycles of 95 °C for 10 s, and 55 °C for 30 s) to allow efficient multiplexing with interchangeable component target ‘cassettes’. Targets included a 118 base-pair conserved region on the 5′ end of *A. phagocytophilum msp2* and a 79 base-pair region nearer to the 3′ end of *E. chaffeensis* single copy *vlpt* (Table 1). Human beta-actin (*ACTB*) was used as an internal amplification control. All primers and probes were synthesized by Integrated DNA Technologies (IDT, Coralville, IA, USA). To determine analytical sensitivity, DNA from cultured *A. phagocytophilum* and *E. chaffeensis* was amplified, cloned, and plasmid quantity determined to develop a standard curve (10^7^, 10^5^, 10^3^, 10^1^ and 10^0^ copies/μL).

The IRBs of Johns Hopkins Medicine, the Duluth Clinic/St. Mary’s Hospital (Duluth, MN, USA), St. Luke’s Hospital (Duluth, MN, USA), New York Medical College (Valhalla, NY, USA), the New York State Department of Health (Albany, NY, USA) and the University of Texas Medical Branch (Galveston, TX, USA) reviewed and either approved the research or declared it exempt.

DNA was prepared from 200 µL of EDTA anti-coagulated blood (Qiagen DNeasy Blood and Tissue kit, Qiagen Inc., Valencia, CA, USA or the Purelink Pro96 Genomic DNA Purification kit, Invitrogen, #K182104A) and resuspended in 200 µL of water. Multiplex PCR was performed with the BioRadiCycler’s iQ5 Multicolor Real-Time PCR Detection System for 96 well plates or the C1000 Thermal Cycler’s CFX™384 Real-Time PCR Detection System (BioRad) for 384 well plates. For the iQ5, each reaction included 2 µL of DNA, 12.5 µL of IQ Multiplex Powermix (BioRad), and primers and probes at a final concentration of 10nM each. For the CFX, each reaction included 5 µL of DNA, 5 µL of IQ Multiplex Powermix (BioRad), and primers and probes at a final concentration of 200 µM each. Each run included duplicates of template, plasmid standards, positive and negative DNA controls, and no template controls. Standard two-step qPCR was performed with initial denaturation at 95 °C for 3 min, 40 cycles of denaturation at 95 °C for 10 s, and annealing/extension at 55 °C for 30 s.

Results were automatically analyzed using a threshold for each probe–fluor pair and BioRad’s baseline-subtract-curvefit algorithm to normalize each run and manually inspected for quality with baselines corrected when necessary. We required positives to be positive in duplicate. Endpoint analysis was accomplished using the BioRad IQ5 or CFX384 software. RFU were averaged over the last 5 cycles and tolerance calculated as a percentage of the difference between the highest and the lowest average RFU for the plate. Potential cut-offs were determined by adding a tolerance to the negative controls’ average RFU and receiver operator characteristic (ROC) curves were constructed to choose optimal cut-offs. Starting quantity means were determined for duplicate positive samples to quantify bacterial load. Final bacterial loads were adjusted to account for 100 copies of *msp2* in the *A. phagocytophilum* genome and for a single *vlpt* copy in the *E. chaffeensis* genome.

## Figures and Tables

**Table 1:** Primers and probes used for singleplex and multiplex PCR for *A. phagocytophilum* and *E. chaffeensis*.

Target Pathogen	Target Gene	Sense Primer	Anti-Sense Primer	Probe
*Anaplasma phagocytophilum* (singleplex)	*msp2*	GAAGATGAWGCTGATACAGTA	CAACHGCCTTAGCAAACT	FAM-TTATCAGTCTGTCCAGTAACA-TAMRA
*Anaplasma phagocytophilum* (multiplex)	*msp2*	GAAGATGAWGCTGATACAGTA	CAACHGCCTTAGCAAACT	Cy5-TTATCAGTCTGTCCAGTAACA-BHQ1
*Ehrlichia chaffeensis*	*vlpt*	CTAATTCTGATTTACACGAGTCTTC	GCATCATCTTCGAATTGAACTTC	TAMRA-TTGAGTTACCTGGTCC-BHQ1
